# Transmittance Control of a Water-Repellent-Coated Layer on a Tensioned Web in a Roll-to-Roll Slot-Die Coating System

**DOI:** 10.3390/polym13224003

**Published:** 2021-11-19

**Authors:** Seongyong Kim, Minho Jo, Jongsu Lee, Changwoo Lee

**Affiliations:** 1 Department of Mechanical Design and Production Engineering, Konkuk University, Seoul 05029, Korea; arsen6788@konkuk.ac.kr (S.K.); als8080@konkuk.ac.kr (M.J.); 2 Department of Printed Electronics Engineering, Sunchon National University, Suncheon 57922, Korea; jslee0505@scnu.ac.kr; 3 Department of Mechanical Engineering, Konkuk University, Seoul 05029, Korea

**Keywords:** contact angle, roll-to-roll, slot-die coating, transmittance, water-repellent coating

## Abstract

Solar cells are important alternatives to fossil fuels for energy generation in today’s world, where the demand for alternative, renewable sources of energy is increasing. However, solar cells, which are installed outdoors, are susceptible to pollution by environmental factors. A solution to overcome this limitation involves coating solar cell surfaces with functional coatings. In this study, we propose a transmittance control method for a tensioned web in a roll-to-roll, transparent, water-repellent film coating. First, we analyzed the effects of process conditions on the transmittance and contact angle of the transparent water-repellent film during roll-to-roll slot-die coating. It was confirmed that the tension was the most dominant factor, followed by the coating gap. Through the tension control, the transmittance was changed by 3.27%, and the contact angle of the DI water was changed by 17.7°. In addition, it was confirmed that the transmittance was changed by 0.8% and the contact angle of DI water by 3.9° via the coating gap control. Based on these results, a transmittance prediction model was developed according to the tension and coating gap, and was then verified experimentally. Finally, a water-repellent film with a high transmittance of 89.77% was obtained using this model.

## 1. Introduction

Fossil fuels are still widely used in various fields, but they have limitations in their reserves. To remedy this issue, active research is currently being conducted on various alternative fuel sources. Solar cells are one of the major alternatives to fossil fuels. Solar cells, by nature, are installed outdoors, where they are exposed to sunlight for a long time; in the case of such outdoor installations, the surface of the solar cell is susceptible to pollution by snow and rain, and is exposed to severe environmental conditions. Such pollutants block the incidence of sunlight on the solar cell, thus decreasing its efficiency. Therefore, research has been conducted on the application of a self-cleaning coating to minimize the reflection of sunlight on the outer shell glass of the cell and effectively eliminate surface contamination, increasing the cell’s efficiency [1,2,3,4,5]. There are various antireflective coatings that minimize the reflection of sunlight on the glass surface of solar cells, which can be distinguished based on their implementation: those with multilayer thin films involving the use of vacuum deposition processes [6,7]; those using silica nanoparticles synthesized via sol–gel processes [8,9,10,11], and those with a periodic nanostructure of ≤300 nm [12,13]. In the case of solar cells, antireflective coatings can be applied to a large surface via a low-cost process.

As such, antireflective coatings using silica nanoparticles, which are capable of self-cleaning, are effective. As a representative nanoparticle, TiO_2_ has a high oxidizing power, and is a widely used environmental purification catalyst owing to its performance in the degradation reaction of hard-to-degrade contaminants. However, increasing the amount of TiO_2_ in the functional coating layer increases the reflection of light, which leads to a low optical transmittance [2]. Many studies have been conducted on the functional coatings of SiO_2_ thin-film structures capable of high optical transmittance for the surface of solar cells.

Most hydrophobic coatings are manufactured via processes such as dip-coating, micro 3D printing, and pulsed laser deposition; however, transparent hydrophobic films produced via such methods (3D printing, laser deposition, etc.) are unsuitable for mass production [14].

The roll-to-roll continuous process involves the formation of a web via tension and meander control using a flexible film [15]. Functional films can be manufactured via continuous processes such as gravure printing, blade coating, and slot-die coating [16,17]. The slot-die coating process is widely used because its thin film thickness can be predicted and controlled using a process variable. Research has been carried out on the governing equation of slot-die coating process by L.E. Scriven and M.S. Carvalho [18], and recently, research on functional film coatings based on the slot-die coating process has been conducted. C. Lee investigated electrolyte layer coating of a solid oxide fuel cell via a slot-die coating process based on the roll-to-roll continuous process, and examined the preventive measures against cracking between the coating processes of brittle materials [19,20,21,22,23]. Another study was conducted to reduce the pinned-edge phenomenon in the coating layer while using high-viscosity fluid-based coatings [24]. Vak conducted a study on the photoactive layer of OPV cells using a template-controlled slot-die [25].

In this study, we propose a transmittance control method for tensioned webs. We analyzed the effects of the process conditions on the transmittance and contact angle of the transparent water-repellent film coating during roll-to-roll slot-die coating. In the roll-to-roll continuous process, the transmittance was affected by the tension and the coating gap, which was verified experimentally. Additionally, a transmittance prediction model was developed based on tension and coating gap, and the performance of the prediction model was verified experimentally.

## 2. Materials and Experiments

### 2.1. Nanoparticles and Hydrophobic Solution

**Figure 1:**
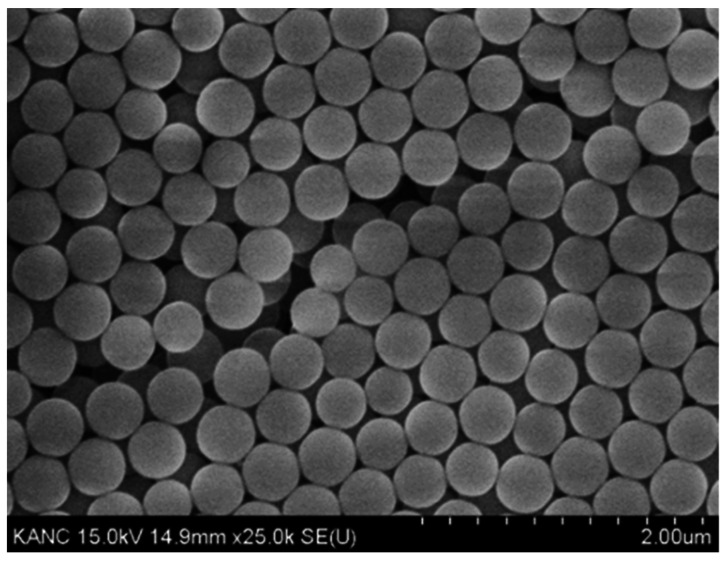
Scanning electron microscopy image of the nanoparticles (SiO_2_).

Silica nanoparticles were synthesized via the sol–gel process, and the composition ratio for synthesizing these particles was as follows: The reagents used for synthesis included tetraethyl orthosilicate (TEOS 95%), ethanol (ethyl alcohol, three thousand, 99%), ammonia solution (ammonium hydroxide solution, 28%) obtained from Sigma-Aldrich, St. Louis, MA, USA, and distilled water. To synthesize the silica nanoparticle solution, 10 mol deionized (DI) water and 2 mol ammonium hydroxide solution were added to 1 L of ethanol; this was followed by stirring for 1 h, the addition of 0.28 mol TEOS, and subsequent stirring for 3 h. Figure 1 shows the scanning electron microscopy images of the prepared nanoparticles, which were fabricated with a uniform size of 150 nm.

The hydrophobic solution was prepared by mixing silica nanoparticles prepared based on TEOS with 1H, 1H, 2H, 2H-perfluorodeclytriethoxysilane (Sigma-Aldrich, St. Louis, MA, USA) and ethanol [26,27]. The composition ratio and physical properties of the hydrophobic coating solution are presented in Table 1 and Table 2 respectively.

### 2.2. Experiments

**Figure 2:**
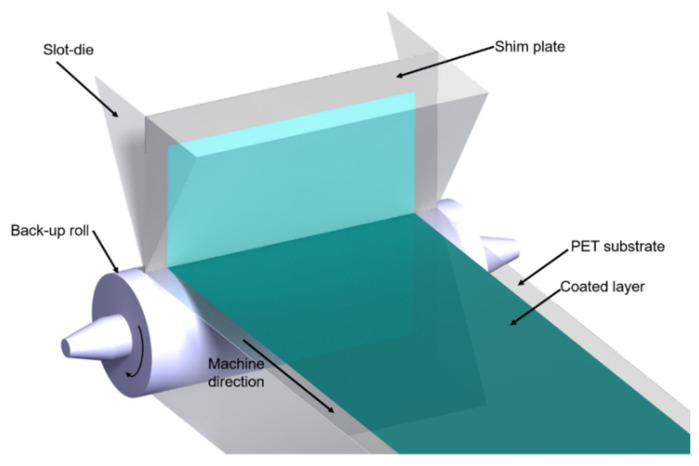
Schematic diagram of the slot-die coater system.

Transparent hydrophobic films were fabricated via slot-die coating. A schematic diagram of the slot-die coating process is shown in Figure 2, and the performance and transmissibility of the transparent hydrophobic film were determined as a function of the surface roughness. To minimize the effect on the thickness, the flow rate—which affects thickness the most—was fixed, and the experimental case was determined using a full factorial design.

The experimental cases are shown in Table 3; the selected process conditions were determined according to the coating gap and tension. The coating gap was a variable for selecting the minimum thickness of the coating in the visco-capillary model based on the capillary numbers, and the flow rate was fixed because it has the highest effect on the coating layer thickness. Tension is a process variable that acts as the traction force of the film in the roll-to-roll continuous process. The applied tension changes the film’s surface energy, changing the contact angle between the film and the coating solution, thus affecting ink spreadability [28,29].

**Figure 3:**
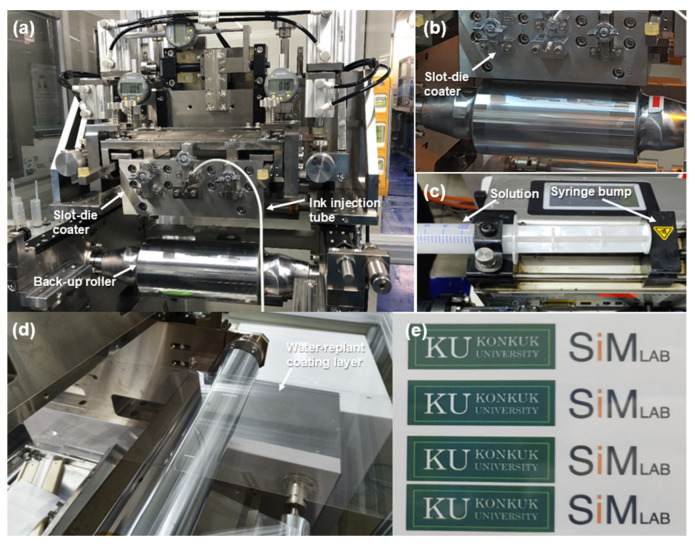
Schematic diagram of slot-die coater system: (**a**,**b**) Slot-die coater and back-up roll, (**c**) syringe pump, (**d**) coating layer, and (**e**) transmittance of water-repellent film.

Figure 3a shows the coating equipment used for the transparent water-repellent film coating, Figure 3b shows the slot-die coater and back-up roll, Figure 3c shows the syringe pump for injecting the water-repellent coating solution, and Figure 3d shows the uniform coating layer; it was confirmed that edge waves and pin holes do not occur. Figure 3e shows the transmittance of the coating film.

## 3. Results

**Figure 4:**
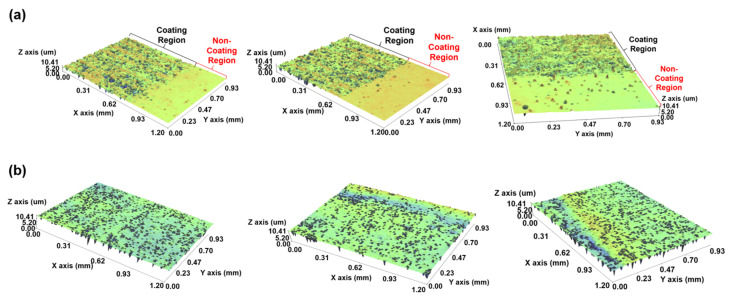
Interferometer measurement results of the transparent water-repellent film: (**a**) thickness and (**b**) surface roughness.

Figure 4 shows the results of measuring the thickness and surface roughness of the transparent water-repellent coating layer with an interferometer.

The flat surfaces and artefact surfaces indicate the non-coating region and coating region, respectively. It can be seen that the surface roughness of the transparent water-repellent coating layer changes according to the process conditions. The surface roughness decreased with increases in the tension and the coating gap. With increasing tension, the surface energy of the material increased, thereby increasing the spreadability of the coating solution and reducing the surface roughness of the coating layer. The coating gap is associated with the formation of stable beads [19] in the coating layer, and as the coating gap increases, the beads’ stability increases, leading to a decrease in the surface roughness. Depending on the process conditions, surface roughness varies with the particle size and viscosity of the solution, making its theoretical prediction impossible. Therefore, surface roughness was predicted by deriving a prediction model from a regression model for water-repellent coating cases only. Table 4 analyzes the effects of the tension and coating gap on the surface roughness, based on ANOVA [29,30,31,32]. F-values indicate the degree to which each term affects the response, while *p*-values are an index for determining the effective factor. As a result of the analysis, it was confirmed that both the tension and the coating gap were effective factors, and the alternating effect was also effective. Based on the results of the analysis, a regression model for predicting surface roughness based on process conditions was derived. The surface roughness prediction model according to the process conditions is given in Equation (1):

Surface roughness = 91.01 − 8.156 × Tension − 0.0139 × Coating gap − 0.000175 × Tension × Coating gap(1)

The R-squared value of the regression model was 99.68%, and it was confirmed that the regression model showed very high accuracy.

**Figure 5:**
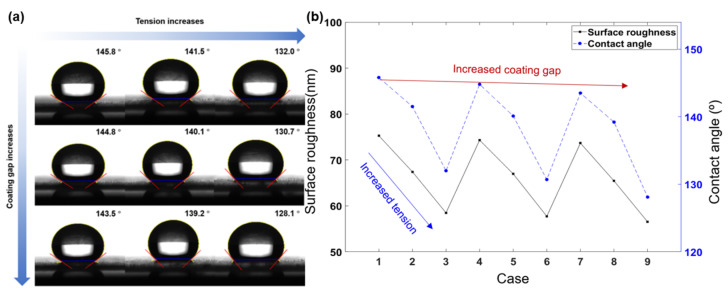
(**a**) Measurement image of DI water contact angle according to the changes in the tension and coating gap. (**b**) Surface roughness and contact angle according to the changes in the tension and coating gap.

**Figure 6:**
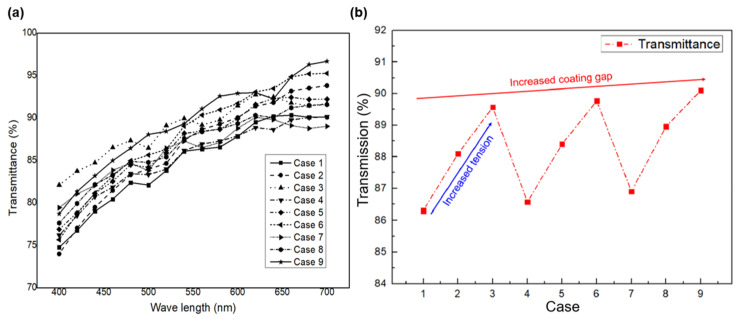
(**a**) Transmittance change in the visible light range. (**b**) Representative transmittance in the visible light range, according to changes in the tension and coating gap.

Figure 5a indicates the hydrophobic performance of the transparent hydrophobic film for each process condition, while Figure 5b indicates the contact angle of the DI water droplet according to the changes in the tension and coating gap. Figure 6a shows the change in transmittance in the visible light region, while Figure 6b shows the representative transmittance in the visible light region, according to the changes in the tension and the coating gap. To measure the hydrophobic performance of the coating, transmittance was measured in the visible region in order to measure the contact angle (Phoenix-300 Touch, Suwon, Korea) and the transmittance based on DI water (Figure 5). The range of transmittance was 86.3–90.1%, which shows a variation of 3.8% for the all of the experimental cases. Cases 1–3, 4–6, and 7–9 indicate the changes in transmittance for each increase in tension, and cases 1, 4, and 7 indicate the changes in transmittance for increased coating gap. The hydrophobic performance showed a variation of approximately 17.7° in the range of 128.1–145.8°. Table 5 presents the transmittance and water-repellent performance according to the process conditions. Tension had a greater effect than the coating gap on hydrophobic performance; as the tension increased, the contact angle decreased. When the tension increased by 2 kgf, the contact angle changed by up to 15.4°. As the coating gap increased, the contact angle of DI water decreased, and when the coating gap increased by 100 µm, the contact angle changed by 3.9°. With respect to the process variables affecting transmittance, tension had a greater effect than the coating gap, and when the tension increased, the transmittance increased at a wavelength of 550 nm; when the tension increased by 2 kgf, the transmittance increased by up to 3.27%.

When the coating gap increased by 100 µm, the transmittance increased by 0.8%. As a result of ANOVA of the transmittance, the *p*-value of the tension was 0.0007, while for the coating gap it was 0.01, indicating that the tension had a greater effect than the coating gap. This is because the spreading properties of ink change as the tension increases, and the surface roughness changes. From the results, it was confirmed that hydrophobic performance and transmittance could be controlled without changing the material, by controlling the tension-applied web in the roll-to-roll slot-die coating process.

## 4. Reflectance Model

### 4.1. Assumption

- Light is incident vertically, and there is no decrease in its transmittance due to the angle of incidence;
- The absorbance of the substrate and the coating layer is negligible [30];
- It is assumed that there is no air gap between the substrate and the coating layer, and no decrease in the transmittance from the gap.

### 4.2. Transmittance Model

**Figure 7:**
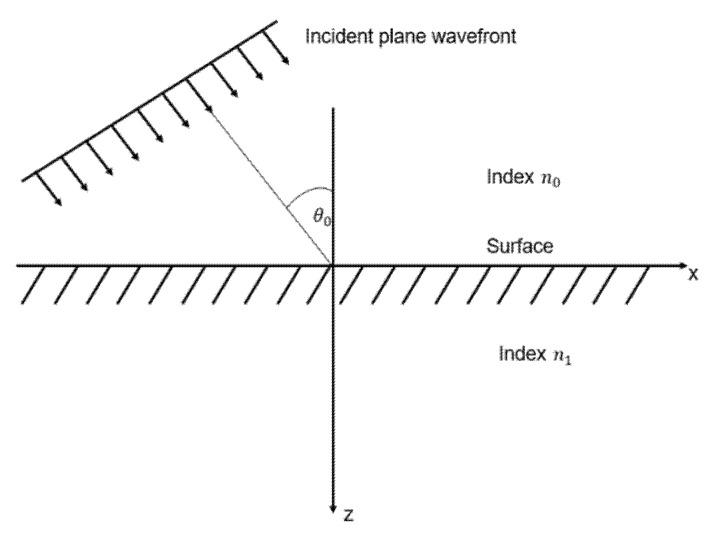
Schematic diagram of light incident on the surface.

Figure 7 reveals a schematic diagram of the light incident on the surface. The refractive index n of materials can be represented as the ratio between the magnetic component amplitude (*H*) and electric component amplitude (*E*) in the corresponding area.

The refractive index (*n*_0_) of air can be determined as follows:

(2) $$n_{0}=\frac{H_{0}}{E_{0}}$$

and the refractive index (*n*_1_) of the substrate can be determined as follows:

(3) $$n_{1}=\frac{H_{1}}{E_{1}}$$

where *H* is the amplitude of the tangential component of the magnetic field—that is, the field parallel to a boundary—and *E* is the amplitude of the tangential component of the electric field—that is, the field parallel to a boundary.

According to Snell’s law, the amplitude reflection coefficient (ρ) of the system is:

(4) $$\rho =\left(\frac{n_{0}-n_{1}}{n_{0}+n_{1}}\right)$$

Here, the reflectance ($R_{0}$) can be represented as $\rho^{2}$

The reflectance of the light from the surface when the light incident from air is transmitted through the substrate with a soft surface is expressed as:

(5) $$R_{0}={\left(\frac{n_{0}-n_{1}}{n_{0}+n_{1}}\right)}^{2}$$

The total integrated scattering by surface roughness can be determined as follows:

(6) $$TIS={\left(\frac{4\pi \sigma cos\theta }{\lambda }\right)}^{2}$$

where *σ* is the surface roughness (RMS) of the substrate, *θ* is the angle of incidence between the light and the substrate, and *λ* is the wavelength of the light.

The *TIS* from Equation (7) represents the amount of incident light at a given angle scattered by the surface roughness. At this time, when light is vertically incident, as shown in Figure 7, $\theta_{0}$ becomes 0, so *cosθ* = 1, and the TIS for the vertically incident light is expressed as:

(7) $${\left(\frac{4\pi \sigma n}{\lambda }\right)}^{2}$$

The change in the reflectance (Δ*R_s_*) of the light scattered by the surface roughness can be represented as:

(8) $$\Delta R_{s}≅R_{0}*{\left(\frac{4\pi \sigma n}{\lambda }\right)}^{2}$$

The reflectance of the substrate considering the surface roughness in the air (*R*_0_) can be expressed as [31]:

(9) $$R=R_{0}+\frac{\Delta R_{2}}{10}$$

**Figure 8:**
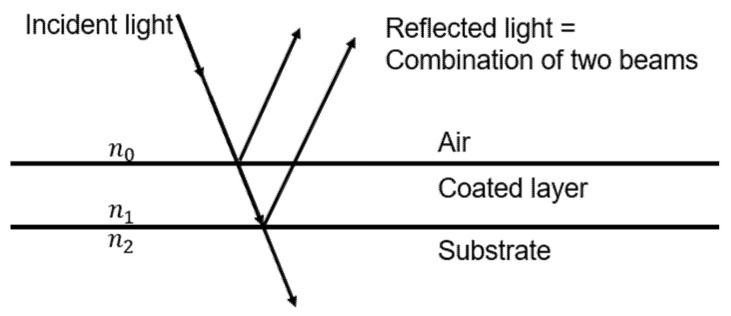
Schematic diagram of the reflection of light from a multilayer film.

Figure 8 shows the reflectance of light on a thin film with a coating layer. A schematic of the substrate and the coating layer in the air is shown, as above. The amplitude reflection coefficient on the interface between the air and the coated layer (*r*_10_) is as follows:

(10) $$r_{10}=\frac{n_{0}-n_{1}}{n_{0}+n_{1}}$$

The amplitude reflection coefficient between the coated layer and the substrate (*r*_21_) is as follows:

(11) $$r_{21}=\frac{n_{1}-n_{2}}{n_{1}+n_{2}}$$

In the interface between the coating layer and the substrate (Boundary b), the magnetic component amplitude (*H*) and electric component amplitude (*E*) can be represented as positive (incident direction) and negative (direction of reflection), with reference to the *z*-axis, and can be determined as follows:

(12) $$E_{21}=E_{21}^{+}+E_{21}^{-}$$

and:

(13) $$H_{21}=n_{1}E_{21}^{+}+n_{1}E_{21}^{-}$$

where $E_{12}^{+}$ is the amplitude of the tangential component of the electric field when it is positive, $E_{12}^{-}$ is the amplitude of the tangential component of the electric field when it is negative, $H_{12}^{+}$ is the amplitude of the tangential component of the magnetic field when it is positive, and $H_{12}^{-}$ is the amplitude of the tangential component of the magnetic field when it is negative. Subscripts 1 and 2 represent the substrate and the nano-thin film material, respectively.

Each component can be expressed as follows:

(14) $$E_{21}^{+}=\frac{1}{2}\left(\frac{H_{2}}{n_{1}}+E_{2}\right)$$

(15) $$E_{21}^{-}=\frac{1}{2}\left(-\frac{H_{2}}{n_{1}}+E_{2}\right)$$

(16) $$H_{21}^{+}=n_{1}E_{21}^{+}=\frac{1}{2}\left(H_{2}+n_{1}E_{2}\right)$$

(17) $$H_{21}^{-}=-n_{1}E_{21}^{-}=\frac{1}{2}\left(H_{2}-n_{1}E_{2}\right)$$

The light incident to the air is transmitted through the coating layer (Equation (1)); therefore, the optical path within the coated layer is higher than the actual thickness of the coating layer. In this case, the phase difference (*iφ*_r_) should be considered.

Here:

(18) $$i\varphi_{r}=\frac{4\pi dncos\theta }{\lambda }$$

and in case of vertically incident light, it is given as:

(19) $$i\varphi_{r}=\frac{4\pi dn}{\lambda }$$

where *d* is the thickness of the coated layer.

*E* and *H* of the coating layer can be expressed as follows:

(20) $$\begin{array}{c} E=E_{20}^{+}+E_{20}^{-}=E_{2}\left(\frac{e^{i\varphi_{r}}+e^{-i\varphi_{r}}}{2}\right)+H_{2}\left(\frac{e^{i\varphi_{r}}+e^{-i\varphi_{r}}}{2n_{1}}\right)\\ \;\;\;=E_{2}cos\varphi_{r}+H_{2}\frac{isin\varphi_{r}}{n_{1}} \end{array}$$

(21) $$\begin{array}{c} H=H_{20}^{+}+H_{20}^{-}=E_{2}n_{1}\left(\frac{e^{i\varphi_{r}}+e^{-i\varphi_{r}}}{2}\right)+H_{2}\left(\frac{e^{i\varphi_{r}}+e^{-i\varphi_{r}}}{2}\right)\\ \;\;\;=E_{2}in_{1}cos\varphi_{r}+H_{2}cos\varphi_{r} \end{array}$$

Here, each component is represented as the product of Equations (14)–(17) and (19), as follows:

(22) $$E_{20}^{+}=E_{21}^{+}e^{i\varphi_{r}}=\frac{1}{2}\left(\frac{H_{2}}{n_{1}}+E_{2}\right)e^{i\varphi_{r}}$$

(23) $$E_{20}^{-}=E_{21}^{-}e^{i\varphi_{r}}=\frac{1}{2}\left(-\frac{H_{2}}{n_{1}}+E_{2}\right)e^{i\varphi_{r}}$$

(24) $$H_{20}^{+}=H_{21}^{+}e^{i\varphi_{r}}=\frac{1}{2}\left(H_{2}+n_{1}E_{2}\right)e^{i\varphi_{r}}$$

(25) $$H_{20}^{-}=H_{21}^{-}e^{i\varphi_{r}}=\frac{1}{2}\left(H_{2}-n_{1}E_{2}\right)e^{i\varphi_{r}}$$

The amplitude of the reflection coefficient is obtained by substituting Equations (19)–(21) into Equation (4).

(26) $$\rho =\frac{r_{20}+r_{10}\exp\left(-i\varphi_{r}\right)}{1+r_{20}r_{10}\exp\left(-i\varphi_{r}\right)}$$

When this is applied in Equation (5) to obtain the reflectance *R*_1_ of the coating layer, *R*_1_ can be represented as follows:

(27) $$R_{1}={\left|r_{1}\right|}^{2}=\frac{r_{10}^{2}+2r_{10}r_{20}\cos\left(\varphi_{r}\right)+r_{20}^{2}}{1+2r_{10}r_{20}\cos\left(\varphi_{r}\right)+r_{10}^{2}+r_{20}^{2}}$$

In this case, the change in the reflectance of the coating layer after considering the surface roughness can be represented as follows, using Equations (27) and (8):

(28) $$\Delta R_{1}=R_{1}{\left(\frac{4\pi \sigma n}{\lambda }\right)}^{2}$$

When *R*_1_ and Δ*R*_1_ are applied to Equation (9), the final reflectance can be calculated as in Equation (29):

(29) $$R=\frac{{\left(r_{10}+r_{20}\right)}^{2}}{{\left(1+r_{10}r_{20}\right)}^{2}}+\frac{r_{10}^{2}+2r_{10}r_{20}\cos\left(\varphi_{r}\right)+r_{20}^{2}}{1+2r_{10}r_{20}\cos\left(\varphi_{r}\right)+r_{10}^{2}+r_{20}^{2}}+\frac{\left(\frac{r_{10}^{2}+2r_{10}r_{20}\cos\left(\varphi_{r}\right)+r_{20}^{2}}{1+2r_{10}r_{20}\cos\left(\varphi_{r}\right)+r_{10}^{2}+r_{20}^{2}}\right)*{\left(\frac{4\pi \sigma n}{\lambda }\right)}^{2}}{10}$$

The transmittance can be calculated as in Equation (30).

(30) $$T=1-R=1-\frac{{\left(r_{10}+r_{20}\right)}^{2}}{{\left(1+r_{10}r_{20}\right)}^{2}}+\frac{r_{10}^{2}+2r_{10}r_{20}\cos\left(\varphi_{r}\right)+r_{20}^{2}}{1+2r_{10}r_{20}\cos\left(\varphi_{r}\right)+r_{10}^{2}+r_{20}^{2}}\;+\frac{\left(\frac{r_{10}^{2}+2r_{10}r_{20}\cos\left(\varphi_{r}\right)+r_{20}^{2}}{1+2r_{10}r_{20}\cos\left(\varphi_{r}\right)+r_{10}^{2}+r_{20}^{2}}\right)\;\times \;{\left(\frac{4\pi \sigma n}{\lambda }\right)}^{2}}{10}$$

In Equation (30), *σ* is the surface roughness of the coating layer, and as the surface roughness increases, the transmittance decreases. Then, the change in transmittance as a result of the change in surface roughness was analyzed via process condition control in slot-die coating.

**Figure 9:**
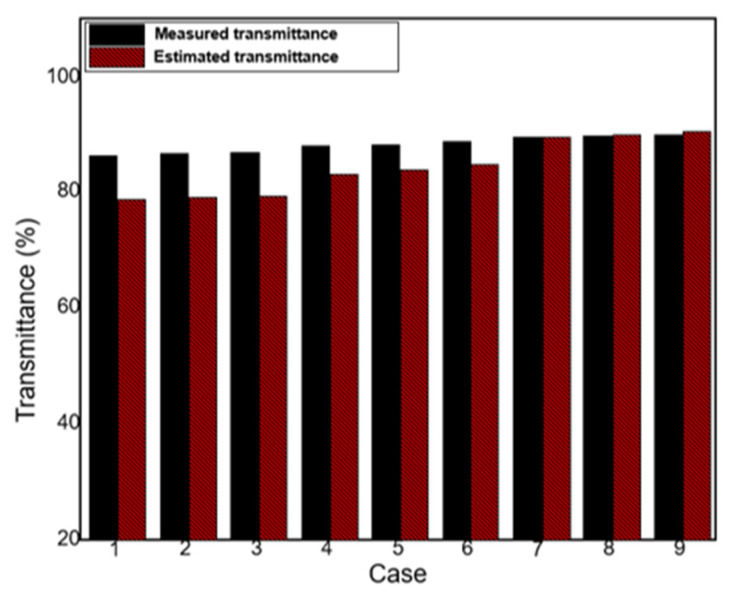
Comparison of the results of the estimated transmittance and the measured transmittance.

**Figure 10:**
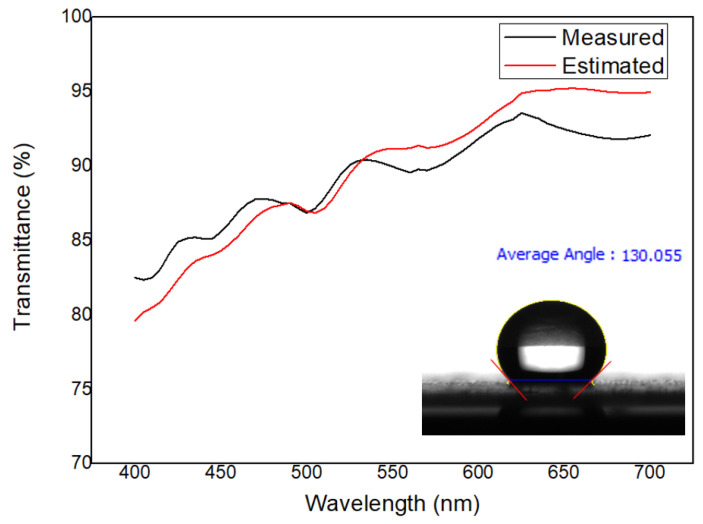
Comparison of the predicted transmittance and measured transmittance of the transparent water-repellent film, based on wavelength.

Next, the performance of the developed model was verified based on measurement results. Figure 9 compares the predicted transmittance values obtained by substituting the predicted surface roughness values based on Equation (1) for those obtained by Equation (30) and the measured transmittance values. The difference between the predicted and measured transmittance at a wavelength of 550 nm was 0.15–7.5%, indicating excellent predictive performance. Figure 10 indicates the transparency of the transparent water-repellent film manufactured under the process conditions derived using the developed model. When the transparent water-repellent film has a contact angle greater than 130°, self-cleaning is possible [33,34,35]. The tension and coating gap required to achieve a film with which the contact angle is greater than 130° were determined to be 3.7 kgf and 150 µm, respectively, using our model. From the figure, one can see that the estimated and experimentally obtained transmittances were 91.17% and 89.77%, respectively, at a wavelength of 550 nm. From these results, the transmittance according to the tension can be estimated, and a high-quality, transparent, self-cleaning film can be obtained based on the model.

## 5. Conclusions

In this paper, we proposed a transmittance control method for tensioned webs. The effects of the process conditions on the transmittance and contact angle of the transparent water-repellent film were analyzed during roll-to-roll slot-die coating. We experimentally verified that the roll-to-roll process conditions affect the transmittance of the water-repellent film, and that the surface roughness of the coating layer affects the water-repellent performance and transmittance of the water-repellent nano-thin film. The water-repellent performance varied from 128.1° to 145.8°, based on the DI water contact angle, while the transmittance at 550 nm varied from 86.3% to 90.1%, based on the changes in the process conditions. Based on these results, a transmittance prediction model was developed. The estimated performance of the developed model was 95.2%, indicating a significantly reliable predictive performance. Finally, a water-repellent film with a high transmittance of 89.77% at 550 nm was obtained using the model. This result suggests that the transmittance according to the tension can be estimated, and a high-quality, transparent, self-cleaning film can be obtained using the developed model.

## Figures and Tables

**Table 1:** Composition ratio of the transparent water-repellent coating solution [27].

Ethanol (wt%)	Nanoparticle (wt%)	1H, 1H, 2H, 2H-Perfluordecyltrietoxsilane (wt%)
91.01	6.09	2.90

**Table 2:** Rheological properties of the transparent water-repellent coating solution.

Weight Percent of Nanoparticles	Viscosity
11.5 wt%	5.8 cP

**Table 3:** Experimental case for performance analysis of the transparent water-repellent film, based on process conditions.

Case	Tension (kgf)	Coating Gap (μm)	Case	Tension (kgf)	Coating Gap (μm)
1	1.7	100	6	2.7	200
2	1.7	150	7	3.7	100
3	1.7	200	8	3.7	150
4	2.7	100	9	3.7	200
5	2.7	150			

**Table 4:** ANOVA of surface roughness based on process conditions.

Properties	*F*-Value	*p*-Value
Tension	360.94	0.0001
Coating gap	7.31	0.0043
Tension × coating gap	0.07	0.0325

**Table 5:** Measurement results for each case.

Case	Tension (kgf)	Coating Gap (μm)	Thickness (μm)	Surface Roughness (Rq) (nm)	Contact Angle (°)	Transmittance (%)
1	1.7	100	0.34	75.28	145.8	86.3
2	1.7	150	0.33	74.31	144.8	86.57
3	1.7	200	0.33	73.66	143.5	86.9
4	2.7	100	0.33	67.42	141.5	88.1
5	2.7	150	0.33	66.97	138.1	88.4
6	2.7	200	0.33	65.43	138	88.96
7	3.7	100	0.34	58.49	132	89.57
8	3.7	150	0.33	57.73	130.7	89.77
9	3.7	200	0.33	56.52	128.1	90.1

## Data Availability

Data presented in this study is available upon request from the corresponding author.
